# Slowing Down the “Magic Bullet”: Encapsulation of Imatinib in Fe-MOF for Cardiotoxicity Reduction and Improvement in Anticancer Activity

**DOI:** 10.3390/molecules29163818

**Published:** 2024-08-12

**Authors:** Weronika Strzempek, Elżbieta Menaszek, Monika Papież, Barbara Gil

**Affiliations:** 1 Faculty of Chemistry, Jagiellonian University, Gronostajowa 2, 30-387 Kraków, Poland; 2 Faculty of Pharmacy, Jagiellonian University Collegium Medicum, Medyczna 9, 30-688 Kraków, Poland; elzbieta.menaszek@uj.edu.pl (E.M.); monika.papiez@uj.edu.pl (M.P.)

**Keywords:** imatinib, metal-organic frameworks, cardiotoxicity inhibition, FeMIL-100, FeMIL-101-NH_2_, lymphoblastic leukemia, drug delivery

## Abstract

Imatinib, a small molecule kinase inhibitor, is used as a cancer growth blocker. However, one of its most serious side effects is congestive cardiac failure. Reducing drug toxicity may be achieved through the use of drug delivery systems. Biocompatible metal-organic framework (MOF) materials, namely FeMIL-100 and FeMIL-101-NH_2_, were employed as potential imatinib carriers. They efficiently delivered the drug as an anticancer agent while minimizing cardiotoxicity. Notably, the release of imatinib from FeMIL-100 was rapid in acidic conditions and slower in pH-neutral environments, allowing targeted delivery to cancer cells. The carrier’s pH-dependent stability governed the drug release mechanism. Two release models—Korsmeyer–Peppas and Weibull—were fitted to the experimental data and discussed in terms of drug release from a rigid microporous matrix. Cytotoxicity tests were conducted on two cell lines: HL60 (a model cell line for acute myeloid leukemia) and H9c2 (a cell line for cardiomyocytes). Overall, the metal-organic framework (MOF) carriers mitigated imatinib’s adverse effects without compromising its effectiveness.

## 1. Introduction

According to estimates from the World Health Organization, cancer is responsible for more than half of all deaths worldwide and is a huge problem and a challenge for public health [1]. Blood cancers, such as leukemia, myeloma, and lymphoma, account for almost 10% of all cases. Leukemias are a group of malignant disorders characterized by an increase in leukocytes in the blood or bone marrow. There are several types and subtypes of leukemia, which vary primarily in pathogenesis, origin (myeloid cells or lymphoid cells), course (chronic or acute), incidence, and death rate. The cause of chronic myeloid leukemia (CML) is the rearrangement of genetic material between chromosomes 9 and 22 in an early stage of myeloid cell formation. These changes lead to the creation of an abnormal BCR-ABL1 gene (most often found on the Philadelphia chromosome) and an oncoprotein: BCR-Abl tyrosine kinase [2]. As a result, there is constant kinase activity, causing abnormality overproduction and reducing sensitivity to apoptotic factors (programmed cell death) [3].

Historically, patients with chronic myeloid leukemia (CML) were treated with busulfan, cytarabine, hydroxyurea, interferon, or a combination of these [4]. Unfortunately, these types of therapies had limitations such as non-specificity, low efficacy, and multiple toxicities. A huge breakthrough in the fight against leukemia was the development of small-molecule tyrosine kinase inhibitors (TKIs), which function as disruptors of the interaction between the BCR-ABL1 oncoprotein and adenosine triphosphate (ATP), resulting in a reduction in tumor cell proliferation. TKIs are not effective in eliminating tumors, but they efficiently slow their growth.

The main representative of TKIs is imatinib mesylate (Gleevec), with a 10 year survival rate slightly higher than 83% [4]. Presently, it is also suggested for use in late CLM relapses [5]. Although imatinib mesylate is highly bioavailable and tolerable, its toxic side effects, such as neutropenia, anemia, vomiting, and diarrhea, are still a problem. One of the most serious side effects is congestive cardiac failure, reported for roughly 7% of patients [4,6,7]. In contrast, Distler and Distler [8] suggested that cardiotoxic side effects occur preferentially in patients with preexisting heart disease, and they undermined the proposed mechanism of cardiotoxicity caused by the ER stress response (unfolded protein response). The clinical doses of many kinase inhibitors are determined based on their toxicity (i.e., the maximum tolerated dose) [9]. Therefore, one of the methods for reducing drug toxicity and preventing the development of drug resistance is the use of drug delivery systems [10]. The encapsulation of imatinib mesylate has already been reported, with nanoparticles, nanoemulsions, polyurethanes, carbon nanotubes, or KIT-5 silica used as carriers [11,12,13,14,15,16,17]. In vivo limitation of cytotoxicity in normal cells (rabbit jugular vein and human internal thoracic vein [18]) and reduced cardiotoxicity (mice hearts [11]) have been reported.

Metal-organic frameworks (MOFs) are commonly used as drug delivery platforms, as summarized in recent reviews [19,20,21,22,23,24]. However, there are only a few examples of using an MOF as an imatinib carrier. Arabbaghi et al. [25] used mesoporous Zn_2_(BDC)_2_(DABCO)-MOF (where BDC stands for 1,4-benzenedicarboxilic acid and DABCO stands for diazabicyclooctane) with a particle size in the range of 25–43 nm and were able to accommodate up to 17.5% wt. of the drug. The authors observed a rapid imatinib release (95% during 30 min) due to the decomposition of the MOF in HCl and PBS buffer, which were used as dissolution media. The release kinetics of the compounds was assessed, but anticancer efficiency or cardiopathy tests were not carried out. Abbasi et al. [26] investigated the uptake and release of imatinib from the Cu_3_(BTC)_2_ (where BTC is benzene tricarboxylate) MOF in two forms: bulk (average particle size of 60.2 nm) and nano (average particle size of 39.5 nm). Imatinib sorption reached 0.082 mmol for 13 g of nano-MOF, which was almost three times higher than that for the activated carbon used for comparison. The authors used ethanol as a dissolution medium. Therefore, the results cannot be directly transferred to the release conditions in the body. The authors did not investigate the cytotoxicity of their materials.

Shahin et al. [27] studied the sorption of imatinib in a shell core composite of Fe_3_O_4_@UiO-66-NH_2_ modified by a reaction between aldehyde groups in glutaraldehyde, MOF amino groups, and pre-adsorbed polyethyleneimine. The authors determined the drug loading efficiency (94%), drug release pH-dependent behavior (at wide range of pH levels (3, 5.5, 7.2, and 9)), and anti-cancer activity against leukemia cell line K562 (with the MTT calorimetric method). Under optimal conditions, imatinib released from the composite had a higher cytotoxicity value than a commercially available drug.

Apart from MOF usage as drug carriers, sorption of imatinib was studied for other applications: sensing (which is directly associated with medical applications), wastewater treatment, and ecological restoration.

Afshar et al. [28] used Zr-MOF (with a terephthalic linker; the specific structure was not named) for the modification of halloysite nanotubes to obtain a highly fluorescent emission at 396 nm, using 270 nm of excitation to determine the IMB concentrations in biological and environmental samples, such as urine or sea water with high recovery (94–99%).

An interesting application of MOFs (binary mixture of TaMOF with CdMOF decorated with gold nanoparticles) was recently proposed by Mir et al. [29], who developed an electrochemical method to measure imatinib in real plasma. Under optimal conditions, the sensor reached a wide region of linear response (0.01–100 μM), a 3 nM limit of detection, and a satisfactory relative recovery percentage (>91%).

The MOF-on-MOF composite MIL-53(Co-Fe)@MIL-53(Ni)/TiO_2_ was used by Ghorbani et al. [30] for adsorption and photodegradation of IMB. The authors determined the optimal sorption conditions, for which a pseudo-second-order kinetic model was fitted, and a high sorption capacity (25.498 mg/g) was obtained.

**Figure 1:**
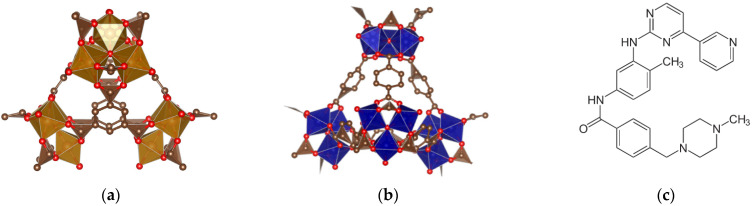
Schematic structures of (**a**) MIL-100 created by VESTA from COD cif file 7102029, (**b**) MIL-101 created by VESTA from COD cif file 4000663, and (**c**) imatinib.

This paper describes the advantages of using iron-based MOFs, FeMIL-100, and FeMIL-101-NH_2_ as potential imatinib carriers and presents their efficacy as anticancer agents with the ability to reduce cardiotoxicity. To establish cytotoxicity, two cell lines are used, namely HL60, a model cell line for acute myeloid leukemia, and the H9c2 cell line (rat ventricular cardiomyocytes). The MIL-100 and MIL-101-NH_2_ structures were chosen because of their structural similarities; they are both built of corner-linked, iron-based supertetrahedra [31]. Their different spatial arrangements of building units causes differences in porosity. Mesoporous cages with sizes of approximately 25 and 29 Å, accessible through microporous windows of roughly 5.5 and 8.6 Å, are formed in an MIL-100 structure [32], and cages of roughly 29 Å and 34 Å, accessible through windows of approximately 12 and 16  Å, are present in MIL-101-NH_2_ [33]. The structures of MIL-100 and MIL-101 are shown in Figure 1.

Both materials are often used as platforms for drug delivery [34,35,36,37] and are non-toxic, as confirmed under in vitro and in vivo conditions [38]. Our previous findings showed that both Fe-MOFs can be used as effective drug carriers, allowing a prolonged release of isoniazid and theophylline [39]. We also proved that FeMIL-101-NH_2_ can be used as a contrast agent for positive and negative magnetic resonance imaging (MRI) [40]. Therefore, it can be expected that Fe-MOF compounds with imatinib could also be applied in theranostics, which is especially important in cancer treatment.

The main objective of this paper is the preparation of non-toxic imatinib@MOF composites able to reduce the most adverse side effect of imatinib therapy (i.e., cardiotoxicity) without reducing the effectiveness of the treatment.

## 2. Results and Discussion

### 2.1. Preparation and Characterization of IMB@FeMIL-100 and IMB@FeMIL-101-NH_2_ Composites

The content of imatinib encapsulated in the studied MOFs was determined through UV-Vis (vide infra) to be equal to 20% wt. for IMB@FeMIL-100 (23% through elemental analysis) and 59% wt. for IMB@FeMIL-101-NH_2_. The procedure of imatinib loading was the same for both MOFs (described in Section 2.3) and was more effective for the latter. There are two possible explanations; more effective encapsulation may be due to bigger channels, especially with larger pore openings in MIL-101 (5.5 and 8.6 Å in MIL-100 versus 12 and 16 Å in MIL-101) or because the presence of the -NH_2_ groups attracted imatinib functional groups. The form of the encapsulated drug and the mode of its interaction with the MOF carriers may be investigated using XRD, FT-IR, and gas adsorption studies.

**Figure 2:**
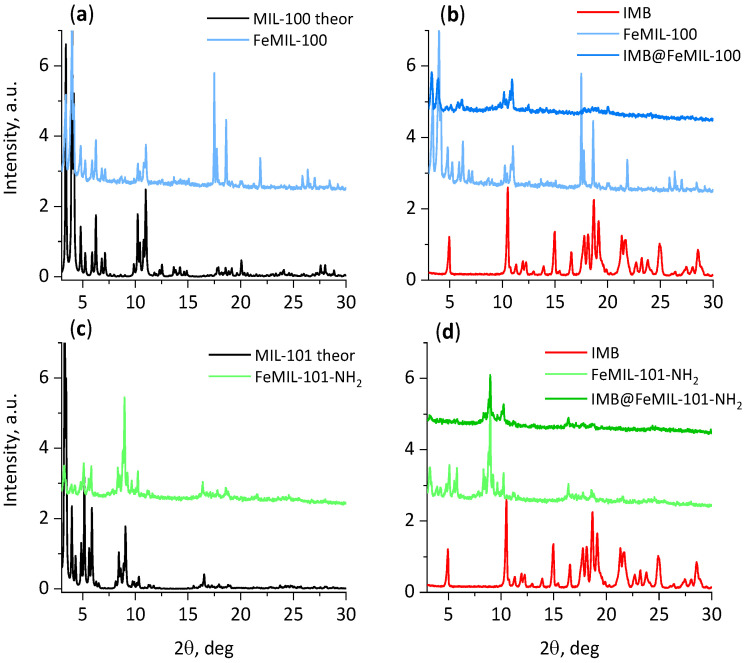
Powder X-ray diffraction patterns of FeMOFs, pure imatinib (IMB), and the IMB@MOFs composites. Theoretical XRD pattern generated by Mercury from cif files 7102029 and 4000663. (**a**) MIL-100, theoretical and experimental, (**b**) imatinib, FeMIL-100, and IMB@FeMIL-101, (**c**) MIL-101 and FeMIL-101-NH_2_, (**d**) imatinib, FeMIL-10-1-NH_2_, and IMB@FeMIL-101-NH_2_.

**Figure 3:**
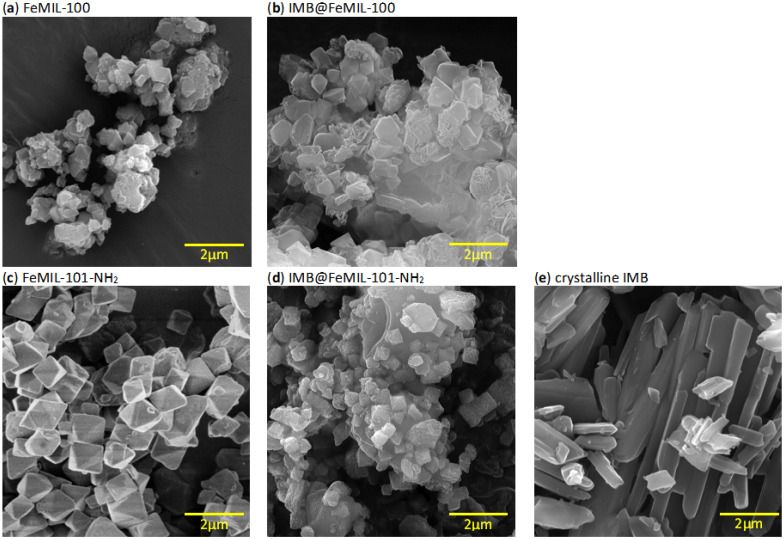
SEM micrographs of (**a**) FeMIL-100, (**b**) IMB@FeMIL-100, (**c**) FeMIL-101-NH_2_, (**d**) IMB@FeMIL-101-NH_2_, and (**e**) pure crystalline IMB.

The XRD patterns of the obtained FeMOF samples are presented in Figure 2. The phase purity and crystalline nature of FeMIL-101-NH_2_ and FeMIL-100 were confirmed by comparing their diffractograms with a simulated PXRD pattern (Figure 2a,c) [23,26]. The variations in intensity of their reflections, especially for MIL-100, can be explained by the possible preferential orientation of its platelet crystals. After imatinib encapsulation, the intensity of the characteristic reflections of both FeMOFs decreased, which may suggest the partial loss of crystallinity of these carriers. Changes in the intensities were different for different 2θ ranges. In the XRD pattern of IMB@FeMIL-101-NH_2_, almost all reflections below 8° 2θ disappeared, while for IMB@FeMIL-100, the intensities at 17–20° 2θ were considerably reduced. Pronounced variations in the powder XRD peak intensities have been observed previously and explained by the changes in symmetry of the host structures upon pore filling [41,42,43]. We have also observed such intensity changes for isoniazid@Fe-MIL-101-NH_2_ [40]. These can be reversible if the drug’s release is not causing carrier decomposition. The absence of degradation of the carriers upon drug incorporation was consistent with crystal morphology preservation, which can be observed in the SEM images (Figure 3), showing that the morphology of the crystals was preserved. The absence of reflections characteristic of crystalline imatinib suggests that the drug was either introduced in an amorphous form or that its crystallites were too small to be detected by a standard XRD instrument (usually below 3–5 nm, caused by X-ray line broadening with a decreasing particle size and lowering of intensity [44]).

Visual inspection of the SEM images provided clues about the location of IMB in composites, namely outside and/or inside the crystals. For pure MOF materials, octahedral crystals of FeMIL-101-NH_2_ and platelet-like crystals of FeMIL-100 can be seen. In both cases, separate crystals of imatinib were not observed. After the introduction of imatinib, the shape of the crystals did not change. The surface of the FeMIL-101-NH_2_ crystals was decorated with small, round particles. It is possible (although due to their small size, it cannot be directly proven) that they are imatinib nanocrystals. This suggests in turn that imatinib was located, at least partially, outside the carrier micropores. Since the entire surface was not covered, but the pores were completely blocked (as confirmed by a sorption measurement; vide infra), imatinib had to also be present inside the pores. In the case of IMB@FeMIL-100, imatinib crystals were not visible, and therefore the drug should have been located mainly inside the pores.

**Figure 4:**
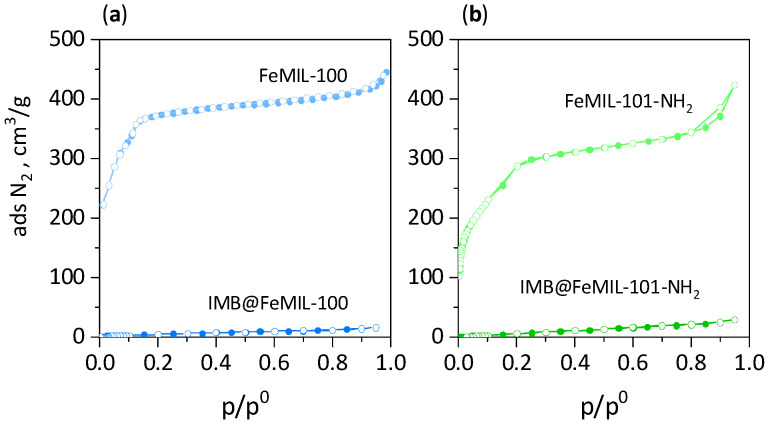
N_2_ adsorption isotherms for FeMOFs and their composites with imatinib IMB@MOFs. (**a**) FeMIL-100 and IMB@FeMIL-100, (**b**) FeMIL-101-NH_2_ and IMB@FeMIL-101-NH_2_.

The porosity of the materials before and after imatinib’s introduction was measured using low-temperature adsorption and desorption of nitrogen. The isotherms obtained for pure FeMOF were representative of microporous materials, with small hysteresis loops closing at p/p^0^ = 0.8. (more pronounced for FeMIL-101-NH_2_), which is typical of intercrystalline mesopores (Figure 4). Two inflection points observed for both MIL structures at p/p^0^ ≅ 0.06 and 0.12 were due to the presence of two types of microporous windows and mesoporous cages of different sizes [32]. After imatinib encapsulation, the composites were almost non-porous, as the drug completely filled or blocked the MOF pores, leaving no accessible pore volume for the nitrogen (Figure 4 and Table 1).

**Figure 5:**
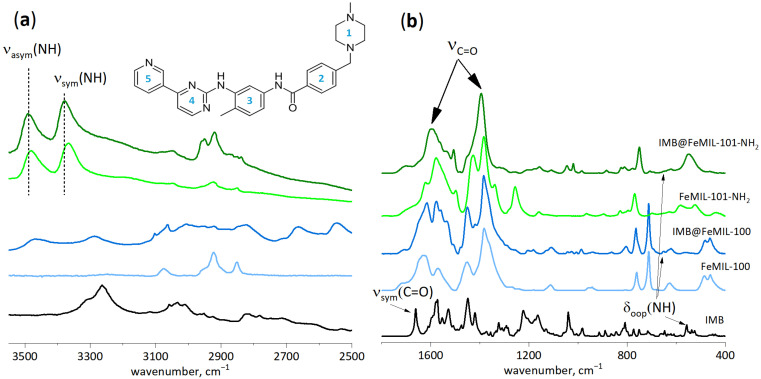
FT-IR spectra of imatinib mesylate, Fe-MIL-100, FeMIL-101-NH_2_, and the IMB@Fe-MIL-100 and IMB@FeMIL-101-NH_2_ composites activated at 100 °C. (**a**) The region of 3550–2500 cm^−1^. (**b**) The region of 1800–400 cm^−1^. The spectra of MOFs and composites were normalized to the same intensity of the maximum of C=O carbonyl groups (1380–1390 cm^−1^). The rings in imatinib structure are numbered (1 to 5) to make the position of functional groups, -NH and -C(O)NH- clear.

The interaction between imatinib and the MOFs was investigated through analysis of the FT-IR spectra of the composites, presented in Figure 5. The obtained IR spectra of the pure MOF materials were consistent with the spectra of the benchmark materials [45,46], with the characteristic maxima of the carbonyl groups occurring at 1680, 1580, and 1395 cm^−1^.

Imatinib, due to the presence of two donors and seven acceptors of H-bonding, may form a variety of hydrogen bonds. This results in the formation of two polymorphic forms of the pure drug, α and β, with the latter being more stable and therefore less desirable for therapeutic use. The FT-IR spectrum of imatinib used for this study (purchased from Sigma Aldrich) indicated that it was a mixture of both polymorphs (Figure 5). The presence of the β form is suggested by the appearance of its maximum at 550 cm^−1^, which is characteristic of δ_N-H_ (bending out-of-plane N-H vibrations), for the N-H located between the second and the third ring (555 cm^−1^ in α form). The presence of the α form was suggested by the maximum of the C=O characteristic of the symmetric stretching vibrations ν_C=O_ (for C=O located between the second and third rings) at 1663 cm^−1^ (1656 cm^−1^ in β form) [47].

In the composites with MOFs, several types of guest–host interactions may appear, considering both the size and complex structure of the imatinib molecule. The confinement effect (i.e., the influence of the MOF’s pore size) and the interaction with functional groups, as well as interaction through van der Waals forces or through the delocalized π electrons of aromatic rings, change both the guest and host structures [48,49] and are not only important for the catalytic properties of porous materials but can influence the stability of the adsorbed species. Two functional groups in imatinib are easy to follow with FT-IR, namely C=O and N-H vibrations of the -C(O)NH-R moiety located between the second and third aromatic rings. For the carrier, the interaction of -NH_2_’s functionality with adsorbed imatinib may be also observed for IMB@FeMIL-101-NH_2_. The positive partial charge located on the amine protons (due to the high electronegativity of nitrogen) allowed for their interaction with the negatively charged fragments of the imatinib molecules.

In both composites, the band characteristic of C=O (from the -C(O)NH-R moiety) was no longer visible, likely being redshifted and obscured by the intense maxima, which is characteristic of MOF carbonyl groups. Another vibration which is sensitive to changes in imatinib conformation is the maximum characteristic of bending out-of-plane vibration δ_NH_ (from the -C(O)NH-R moiety). The position of this maximum was shifted to higher wavenumbers (from 550 to 655 cm^−1^) inside composites, and its shape in the composite spectra (lowered intensity and increased width) also suggests the formation of a hydrogen bond. The redshift of the stretching vibrations and blueshift of the bending vibrations are characteristic of hydrogen bond formation [50]. According to the DFT calculations of Srivastava et al. [47], the IR maxima of other functional groups are less sensitive to the formation of hydrogen bonds and therefore to changes in conformation.

Based on the FT-IR spectra, it can be concluded that imatinib molecules interact with both MOF matrices through both C=O and NH groups, and the conformation of the imatinib present inside the MOF channels is different from the known conformers: α and β. This is consistent with the observed apparent loss of crystallinity of the composites, caused by the spatial disorder of the adsorbed drug. The IR spectra of MOF carriers also changed upon imatinib incorporation, with the most notable changes occurring in the vibrations of the linker carbonyl groups (1300–1700 cm^−1^; Figure 5b). Although they partially overlapped with the C=O vibration of the imatinib, it is still clear that the presence of the drug influenced the vibrations of the carboxylic linker.

For FeMIL-101-NH_2_, the changes in both the asymmetric and symmetric stretching vibrations of the amine N-H linker could be observed (Figure 5a). Both bands were shifted to lower wavenumbers by 16 and 10 cm^−1^, respectively. These values are too small to be associated with hydrogen bond formation [51], but the perturbation is evident, and the presence of imatinib hindered the N-H bond vibration. As both bands were downshifted, this suggests slight bond elongation. Thus, hydrogens (bearing a partial positive charge) are attracted by the partially negatively charged fragments of imatinib (either delocalized aromatic π electrons or free electron pairs localized on electronegative atoms).

### 2.2. Drug Release

In vitro release is the key standard for testing simple drug and matrix composites as well as evaluating and optimizing more complex formulations targeted for specific drug administration methods [52]. Modern carriers of anticancer drugs must fulfill a wide range of requirements. In addition to efficiently encapsulating drug molecules, they should selectively release them into a tumor’s surrounding environment to prevent or at least minimize toxic effects on healthy cells. The extracellular regions of neoplastic cells are more acidic than normal cells, most likely due to poor perfusion, hypoxia, and high metabolic rates, especially in relation to glucose [53]. For this reason, two buffer solutions were used in the release studies. The first one, phosphate buffered saline (PBS), with a pH level of 7.4, has isotonic osmolarity and ion concentrations matching those of the human body. The acidic endosomal buffer (pH = 4.2) was chosen to mimic the acidic pH in the environment of cancerous cells.

**Figure 6:**
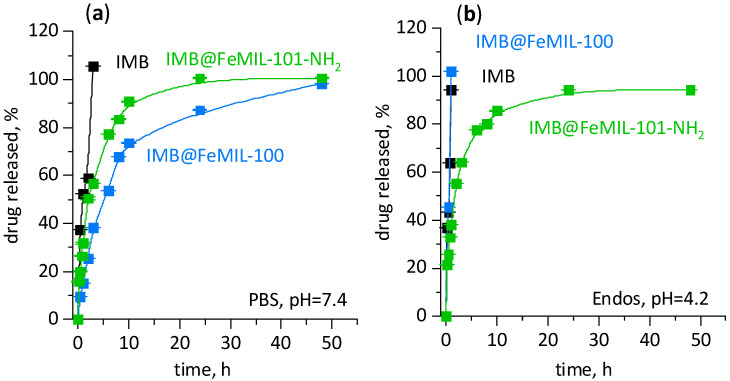
Time-dependent release curves of imatinib (IMB) release from IMB@FeMIL-100 and IMB@FeMIL-101-NH_2_ in (**a**) PBS and (**b**) endosomal buffer. Black line and points correspond to pure IMB, while green is for IMB@FeMIL-101-NH_2_ and blue is for IMB@FeMIL-100.

Crystalline imatinib was released very rapidly to the PBS and endosomal buffer media (Figure 6). Imatinib’s release from IMB@FeMIL-101-NH_2_ was much slower, and the release rate was practically independent of the pH level. The most interesting behavior was observed for IMB’s release from IMB@FeMIL-100, being extremely fast in the acidic medium and much slower in a pH-neutral solution. This is a desirable effect, allowing fast imatinib release in the environment of cancerous cells and much slower release—and therefore intake—by the healthy ones.

Quantitative modeling allows better understanding of the physical phenomena which occur during drug release [54]. Two release models, Korsmeyer–Peppas and Weibull (linearized, Equations (4) and (2), respectively) were fitted to the experimental data. The basic parameters (b and n values, Pearson’s r coefficient, and residual sum of squares (RRS)) are presented in Table 2. Both equations allow modeling a release from both flat and cylindrical porous matrices [55], although originally, they were developed for polymeric ones [56,57]. Additionally, the n value (the release exponent) from the Korsmeyer–Peppas model and the b value (shape parameter) from the Weibull equation may suggest the dominant drug release mechanism.

The exponential Weibull equation (Equation (1)) is

(1) $$f=\frac{M_{i}}{M_{max}}=1-e^{{-\left(\frac{t}{a}\right)}^{b}}$$

(2) $$\ln(-\ln\left(1-f\right))=\mathrm{bln}(t)-\mathrm{bln}(a)$$

where f is the function fitted to the scatter release data, ($\frac{M_{i}}{M_{max}}$) is the fraction of IMB released, t is the time, and a and b are constants.

The exponential Korsmeyer–Peppas equation (Equation (3)) is

(3) $$f=\frac{M_{i}}{M_{max}}=k_{m}t^{n}$$

which can be linearized to Equation (4):

(4) $$\ln\left(f\right)=\mathrm{nln}\left(t\right)+\ln(k_{m})$$

where f is the function fitted to the scatter release data, ($\frac{M_{i}}{M_{max}}$) is the fraction of IMB released, t is the time, k_m_ is a kinetic constant, and n is a release constant.

**Figure 7:**
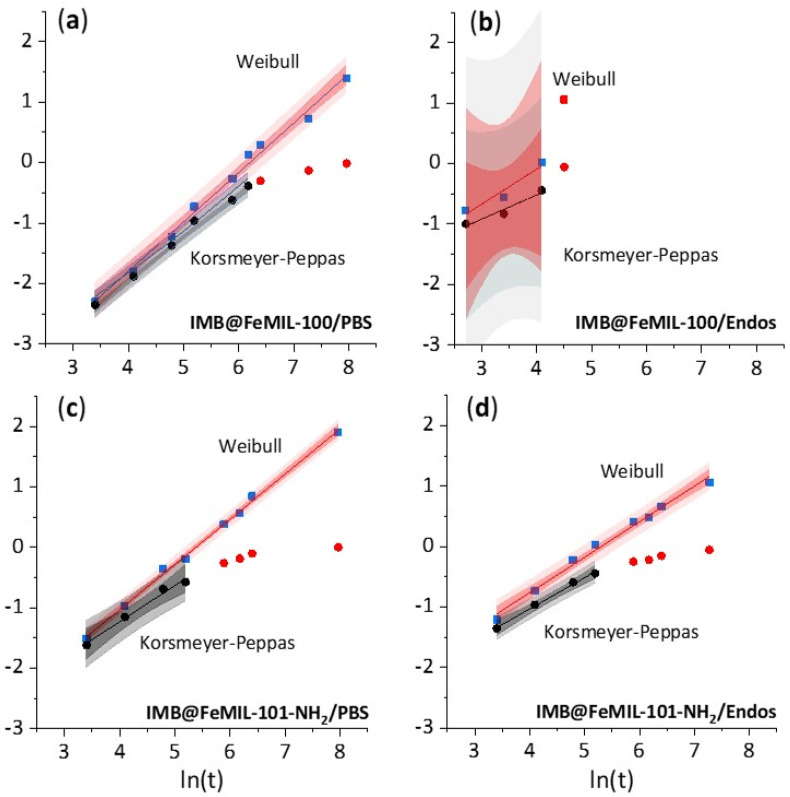
Experimental data from Figure 6 fitted to linearized Weibull and Korsmeyer–Peppas equations (Equations (2) and (4), respectively). Data for the Weibull equation are blue squares, while red bands represent 95% confidence limits (darker red) and 95% prediction limits (lighter red). Data for the Korsmeyer–Peppas equation are black circles (up to approximately 60% release) and red circles (above 60% release) (excluded from fitting), where gray bands represent 95% confidence limits (darker gray) and 95% prediction limits (lighter gray). The ordinate is $ln(-\ln\left(1-f\right))$ for the Weibull model and ln(f) for the Korsmeyer–Peppas model. Imatinib’s release shown (**a**) from IMB@FeMIL-100 to PBS solution, (**b**) from IMB@FeMIL-100 to endosomal solution (Endos), (**c**) from IMB@FeMIL-101-NH_2_ to PBS solution, and (**d**) from IMB@FeMIL-101-NH_2_ to endosomal solution (Endos).

The Korsmeyer–Peppas model is valid for the beginning of release (usually when less than 60% of the drug is released ($\frac{M_{i}}{M_{max}}$ < 0.6)), while the Weibull model may be used for the entire set of release data ($\frac{M_{i}}{M_{max}}$ < 1), as shown in Figure 7.

The MOF channels are cylindrical. Therefore, there are two borderline values for the n parameter in the Korsmeyer–Peppas equation, namely 0.45, dividing Fickian diffusion from anomalous (non-Fickian) transport, and 0.89, dividing the latter from the transport influenced by swelling [55]. In all cases, taking into consideration the standard deviation, 0.45 < n < 0.89, and thus diffusion is not a determining step, pointing to anomalous transport. Deviation from the release mechanism governed by diffusion may be due to several factors. For pure IMB, the pH-dependent solubility has to be taken into account, while for release from a porous matrix, two factors have to be considered: the influence of porosity and possible matrix erosion.

From the Korsmeyer–Peppas equation another parameter may be calculated: the kinetic constant (k_m_) rate of drug release. The k_m_ values were similar for IMB’s release to PBS and Endos (7.76 × 10^−3^ and 2.00 × 10^−3^, respectively). Its release in IMB@FeMIL-100 was of the same order of magnitude (7.59 × 10^−3^), and therefore no change in release kinetics was achieved. When the medium was switched to PBS, the kinetics constant decreased by two orders of magnitude to 1.70 × 10^−5^. The difference in the release rate could not be due only the pH-dependent solubility of the drug because pure IMB was released extremely fast independently of the pH level (km of the same order of magnitude). The effect therefore had to be due to the interaction of the carrier with a different environment. The stability of FeMOFs in water solutions of different pH levels was already explored [58]. Bezverkhyy et al. [59] studied FeMIL-100 degradation in liquid water under reflux at 100 °C and under acidic conditions at ambient temperature. Only partial decomposition was observed at a natural, slightly acidic pH level, while total structure collapse occurred at a neutral pH level, generated by the addition of NaOH. This was also confirmed by Souza [60], who used immersion in pure water to recrystallize poor crystalline FeMIL-100 MOF. FeMIL-101, on the other hand, is much more robust [61].

It may be safely assumed that the fast imatinib release from IMB@FeMIL-100 to Endos was due to matrix decomposition, while the slower release from IMB@FeMIL-100 to PBS and IMB@MIL-101-NH_2_ in both media was due to slow release from the micropores. The smaller pore entrances in MIL-100 compared with MIL-101-NH_2_ were responsible for the slowest release rate. Most probably, additional interaction with amine groups is of secondary importance.

**Figure 8:**
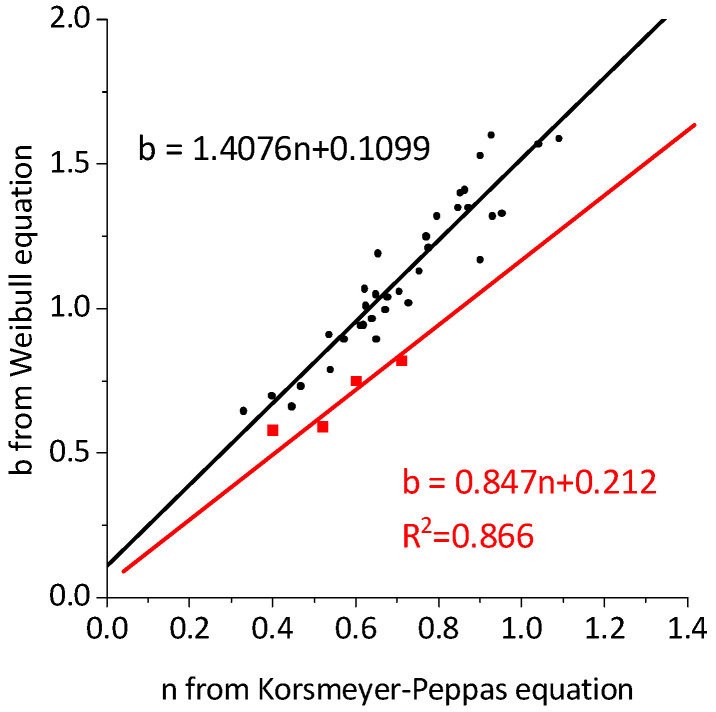
Linear fitting of the estimates for b from the Weibull equation versus the estimates for n from the Korsmeyer–Peppas equation. Red point and line (black points and line are for this work) are from Papadopoulou’s work [62].

The fit of the estimates for b from the Weibull equation versus the estimates for n from the Korsmeyer–Peppas equation gave similar results to the ones observed by Papadopoulou [62] (i.e., b = 1.118n + 0.0388, R^2^ = 0.987 (this work) versus b = 1.4076n + 0.1099, R^2^ = 0.8936 (Papadopoulou)) (Figure 8). As proposed by the authors, b values below 0.75 indicate Fickian diffusion, while a combined mechanism is associated with 0.75 < b < 1. When Papadopoulou’s assumptions were applied to our results, the release mechanism appeared to be dependent on the solution used. For Endos, all b values for IMB@MOFs were below 0.75, indicating diffusion-based release, while b > 0.75 indicated anomalous transport. Since all data used by Papadopoulou were acquired for polymeric matrices, this may suggest that the borderline b values should be modified for rigid, microporous materials.

### 2.3. Cellular Cytotoxicity Studies

Cardiotoxicity is one of the major side effects of imatinib. However, thousands of patients are being treated for leukemia with this drug due to the positive benefit-to-risk ratio. Cellular cytotoxicity was determined based on several tests to provide comprehensive information on the mechanism of imatinib’s influence on neoplastic and healthy cells, human leukemia cells (HL60 line), and cardiomyocytes (undifferentiated H9c2 rat cells). First, viability and proliferation were tested. Then, the extent of cell degradation was assessed, followed by determination of the type of cell death (apoptosis versus necrosis). The influence of the drug on mitochondria and the formation of reactive oxygen species to the extent of oxidative stress were also measured.

#### 2.3.1. Toxicity for Cardiomyocytes

**Figure 9:**
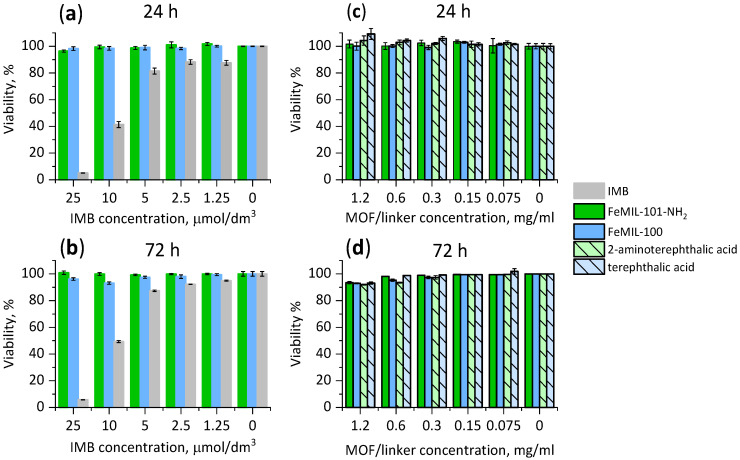
Cardiomyocyte (undifferentiated H9c2 rat cells) viability after 24 h (**a**,**c**) and 72 h (**b**,**d**), determined by PrestoBlue^TM^ test. Cells were incubated with IMB@FeMIL-100, IMB@FeMIL-101-NH_2_, FeMIL-100, FeMIL-101-NH_2_, IMB, 2-aminoterephthalic acid, and terephthalic acid.

Concentration-dependent viability was measured after 24 and 72 h of incubation with the drug-MOF composites (IMB@FeMIL-100 and IMB@FeMIL-101-NH_2_), pure MOFs (FeMIL-100 and FeMIL-101-NH_2_), and organic linkers (terephthalic and 2-aminoterephthalic acid), and we compared the results with the effect of the pure drug (Figure 9).

The drug concentrations (1.25, 2.5, 5, 10, and 25 µmol/dm^3^) were selected based on the cellular response. Initially, a wide range of 0–100 μmol/dm^3^ was applied, and then the range was limited to 25 μmol/dm^3^, which was the lowest concentration, resulting in a significant decrease in viability (both for cardiomyocytes and leukemia cells). Additional literature research indicated that the correct choice was made, and the results could be compared with the available literature data [27,63,64,65].

The viability and proliferation of cardiomyocytes were determined using the PrestoBlue^TM^ test, determining the activity of living cells in the reduction of non-fluorescent resazurin to fluorescent resorufin. Fluorescence (in relative fluorescence units (RFUs)) is proportional to the number of living cells, because damaged and non-viable cells, due to low or absent metabolic activity, generate a lower fluorescence signal than that of healthy cells. Viability is expressed as the percentage of living cells.

For IMB@FeMOF composites, viability was related to the concentration of imatinib, and the protective effect of the carrier increased with the drug concentration. At the highest concentration used (25 μmol/dm^3^), the viability of cardiomyocytes decreased to 5% only when pure IMB was used, while it remained at a rather high level (above 95%) when IMB was incorporated into the MOF. The protective effect of the MOF was already visible for the IMB concentration of 10 μmol/dm^3^. In the case of pure FeMOFs and their linkers, a slight decrease in cardiomyocyte viability was observed only after 72 h, as the viability was reduced by a maximum of 8% (for 2-aminoterephthalic acid, the MOF linker). FeMOFs, independent of their structure, did not cause a cytotoxic effect even at the highest concentration (1.2 mg/mL). An analogous effect was confirmed in our previous studies [40,66]. Other authors also demonstrated that even high doses (220 mg/kg) of FeMOFs (MIL-100, MIL-88A, and MIL-88B-4CH_3_) did not show significant in vivo toxicity after intravenous administration to rats [38].

**Figure 10:**
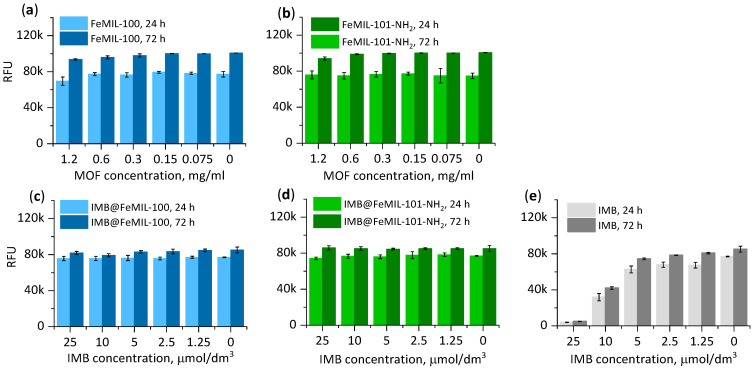
Proliferation, expressed as fluorescence (in relative fluorescence units (RFUs), measured with a PrestoBlue test) as a function of the IMB concentration after 24 and 72 h of cell incubation in the presence of FeMIL-100 (**a**), IMB@FeMIL-100 (**b**), FeMIL-101-NH_2_ (**c**), IMB@FeMIL-101-NH_2_ (**d**), and IMB (**e**).

Based on the fluorescence measured after 24 and 72 h, a marked slowdown in proliferation was observed when the cardiomyocytes (undifferentiated H9c2 rat cells) were incubated in the presence of the pure drug (Figure 10). In the presence of FeMOFs, after 72 h, the number of viable cells was higher than that after 24 h, and the increase was independent of the MOF concentration and almost the same as that in the blank experiment. The same effect was observed for IMB@FeMOF composites, as the proliferation rate did not change compared with the untreated cells even at the highest drug dose of 25 µmol/dm^3^. At the same concentration for the pure drug, proliferation was stopped entirely.

**Figure 11:**
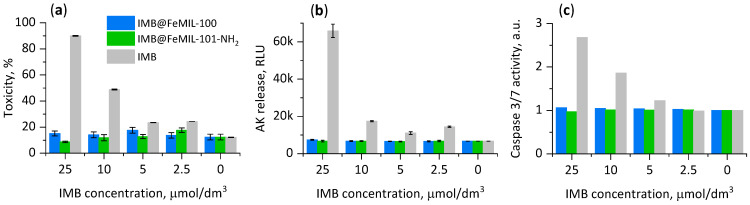
(**a**) Toxicity measured with ToxiLight^TM^ bioluminescent cytolysis kit (H9c2 cells, 24 h). (**b**) Release of adenylate kinase (AK) into the cell culture medium. (**c**) Activity of caspases 3/7.

For the next step, cell damage was measured with a ToxiLight^TM^ bioluminescent cytolysis kit, measuring the release of adenylate kinase (AK), an acytosolic protein released when the cell membrane integrity is compromised. The release of adenylate kinase was measured in culture supernatants and lysate (Figure 11). For both composites, no significant increase in the level of released AK was observed, showing no toxic effect of IMB even if the drug concentration was increased tenfold. In contrast, free imatinib induced cell death in a dose-dependent manner, and the toxicity increased from 24% for the lowest IMB concentration (2.5 μmol/dm^3^) to almost 90% for the highest concentration used (25 μmol/dm^3^).

Since cell death may be due to necrosis or apoptosis, the activity of caspase 3/7, an enzyme involved in the apoptosis process, was also measured (Figure 11c). There was no difference in caspase level for the composites independent of the IMB concentration. For the pure drug, the increase in the caspase level was parallel to the observed release of AK. Therefore, it can be confirmed that the main mechanism of cell death caused by imatinib is apoptosis.

**Figure 12:**
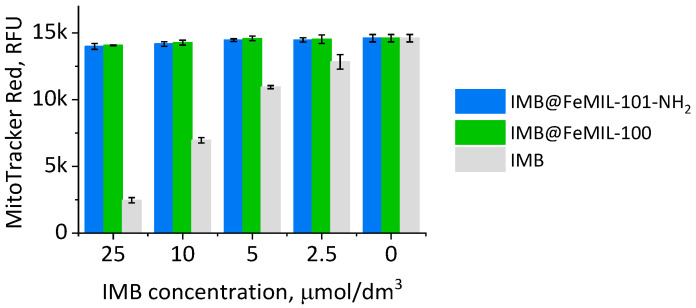
The MitoTracker dye levels (in fluorescence units), showing the dependence of the cardiomyocyte (H9c2 cells) mitochondrial membrane potential as a function of the imatinib (IMB) concentration for the pure drug and IMB@FeMOF composites. Activity measured after 24 h of incubation using the MitoTracker assay.

Kerkela et al. [7,67] identified mitochondria as the main target of imatinib and implicated mitochondrial dysfunction, leading to energy loss, as the crucial factor in cardiotoxicity. The results presented below (Figure 12) support this theory. The dose-dependent collapse of the mitochondrial membrane potential was measured with MitoTracker. The fluorescent dye used in this test actively bound to the mitochondria with preserved membrane potential. For both IMB@FeMOF composites, the level of fluorescence, indicating the presence of living cells with undamaged mitochondria, was practically the same as that for the blank experiment. The presence of pure IMB, even at the lowest concentration, damaged mitochondria, and the effect was dose-dependent.

**Figure 13:**
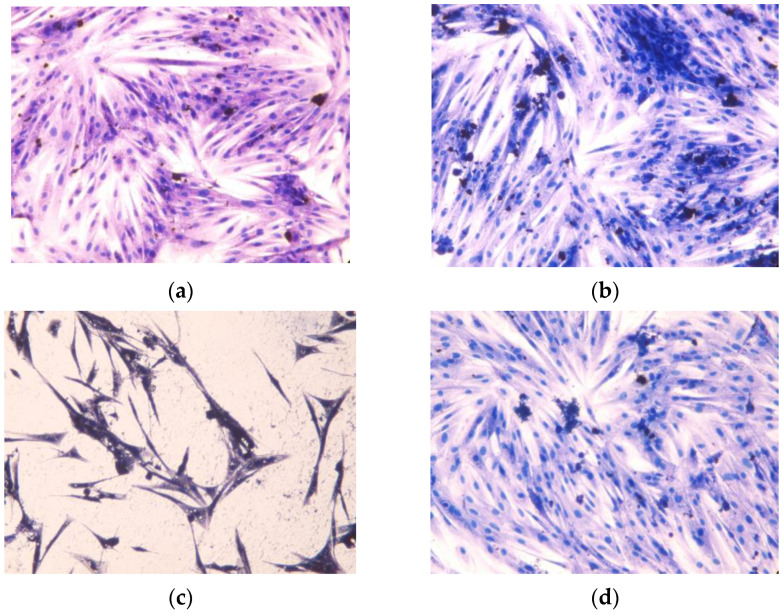
Photomicrographs of H9c2 cardiomyocytes cultured for 24 h in the presence of (**a**) IMB@FeMIL-101-NH_2_, (**b**) IMB@FeMIL-100, and (**c**) IMB, containing 10 μmol/dm^3^ of the drug for each case, together with a control (**d**). Cells were stained with eosin and hematoxylin. The same magnification was used for all graphs.

Loss of normal morphology and cytoplasmic vacuolation were observed for the cells incubated with the free drug (Figure 13). The lack of noticeable morphotic changes and high number of cells incubated with IMB@FeMOFs confirmed that the use of drug carriers eliminated the cardiotoxicity caused by imatinib.

**Figure 14:**
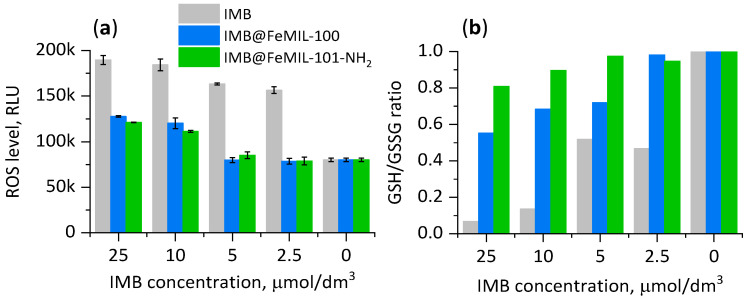
(**a**) Average ROS level for IMB and IMB@FeMOFs in comparison with the control (untreated cells). (**b**) GSH-to-GSSG ratio measured after 24 h of incubation.

Oxidative stress is another factor which potentially leads to cell death, and thus the measurement of reactive oxygen species (ROS) and the ratio of reduced glutathione (GSH) to its oxidized form (GSSG) may be performed to determine the condition of the cardiomyocytes. The glutathione antioxidant system producing reduced glutathione, which is an ROS scavenger, is one of the most basic mechanisms against oxidative stress. Kobara et al. [68] suggested that free imatinib (at a concentration of 10 µmol/dm^3^) leads to cardiomyocyte apoptosis caused by increased levels of ROS in the mitochondria, thus affecting the mitochondrial membrane potential. The production of intracellular reactive oxygen species (ROS) by H9c2 cells was measured using the DCFH-DA probe (Figure 14). The DCFH-DA is internalized within cells in a reduced form, and in the presence of ROS, it is oxidized to its fluorescent form. Thus, the measured fluorescence intensity is proportional to the level of intracellular oxidative stress. The ROS level increased after 24 h of incubation of the cardiomyocytes with free imatinib (increased by a factor of 2.3) and IMB@FeMOF (increased by a factor of 1.5) when compared with the control group. Our data support recently published results showing increased ROS formation in isolated rat ventricular myocytes after the administration of one of the tyrosine kinase inhibitors, sorafenib, a drug with a similar mechanism of action to imatinib [69].

The glutathione levels were determined with a GSH/GSSG Glo assay (Figure 14). In the presence of the pure drug, the GSH/GSSG ratio increased by double at a low imatinib concentration (2.5 µmol/dm^3^) and decreased dramatically for the highest concentration (25 µmol/dm^3^) down to 0.07, with 1 being the baseline GSH/GSSG ratio for untreated cells. This confirmed oxidative stress in the cells, which could eventually lead to cardiomyocyte apoptosis. The imatinib released from IMB@FeMIL-100 and IMB@FeMIL101-NH_2_ had a weaker effect, as the GSH/GSSH ratios measured after the same incubation time (24 h, IMB concentration of 25 μmol/dm^3^) decreased to 0.6 and 0.8, respectively. The higher ROS level for IMB@FeMIL-100 was probably due to the higher release rate of IMB from this carrier (see Table 2).

#### 2.3.2. Toxicity for HL60 Human Leukemia Cells

**Figure 15:**
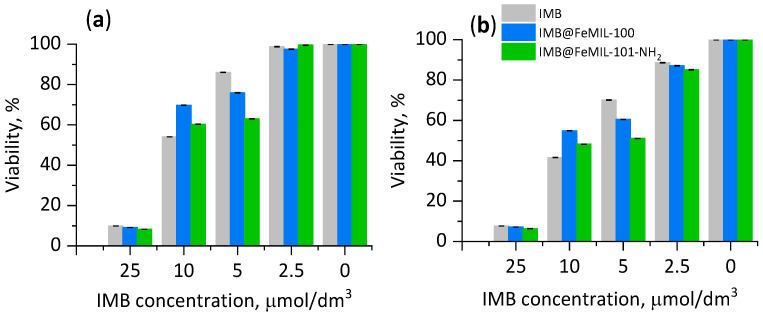
The results of the PrestoBlue^TM^ test. Viability shown for HL60 cells after 24 h (**a**) and 72 h (**b**) of incubation with IMB@FeMIL-100, IMB@FeMIL-101-NH_2_, and IMB.

The toxicity test was performed for human leukemia cells (HL60 line) to show the anticancer effectivity of the IMB@FeMOF composites. The concentration-dependent cell viability and cytotoxic effect induced by IMB@FeMOFs and IMB (as a reference) were measured after 24 h of incubation (Figure 15). At an extremely low imatinib concentration, there was no visible activity, but when the imatinib concentration increased to 10 µmol/dm^3^, leukemia cell viability decreased to 54, 60, and 70%, for IMB, IMB@FeMIL-100, and IMB@FeMIL-101-NH_2_, respectively. For the highest IMB concentration, the viability in all cases was as low as 9%. This shows that imatinib released from FeMOFs has anticancer activity comparable to that of its free form. The cytotoxicity of the same composites toward cardiomyocytes was low, and the viability, independent of the IMB concentration, was above 95%.

**Figure 16:**
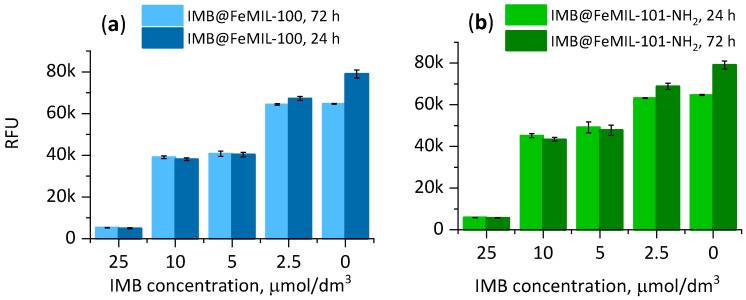
Dependence of fluorescence (in relative fluorescence units (RFUs)) on the IMB concentration, corresponding to the number of viable cells after 24 and 72 h of incubation with (**a**) IMB@FeMIL-100 and (**b**) IMB@FeMIL-101-NH_2_.

The performed viability test confirmed that the imatinib released from both composites, IMB@FeMIL-100 and IMB@FeMIL-101-NH_2_, inhibited leukemia cell proliferation at concentrations greater than 2.5 µmol/dm^3^ and practically stopped proliferation at the highest applied concentration (25 μmol/dm^3^), as shown in Figure 16.

## 3. Conclusions

Two biocompatible metal-organic framework (MOF) materials, FeMIL-100 and FeMIL-101-NH_2_, were employed as potential carriers for imatinib, which can be effective in anticancer therapy without the side effects (i.e., cardiotoxicity). The best results were achieved for FeMIL-100, for which the release of imatinib was rapid in acidic conditions (endosomal buffer, pH = 4.2) and slow in the pH-neutral environment (PBS buffer). This result is promising for the modification of drug delivery, being more efficient for cancer cells, whose extracellular regions are more acidic than normal cells, which are protected from interaction with imatinib.

Imatinib was loaded into FeMIL-100 and FeMIL-101-NH_2_ under the same conditions to compare the drug capacities of both materials. Higher loading was achieved for IMB@FeMIL-101-NH_2_ (59% wt. versus 20% for IMB@FeMIL-100). Higher loading may be possible for FeMIL-100 by optimizing the sorption conditions.

The introduced drug was present in an amorphous form or as extremely small crystals (below the standard XRD detection limits). This is advantageous from the standpoint of drug bioavailability, which is usually higher for amorphous or metastable drug forms. The interaction between imatinib and MOFs was investigated through analysis of the FT-IR spectra of the composites and showed that both the drug and carrier structures were modified by their mutual interaction.

Two release models, Korsmeyer–Peppas and Weibull, were fitted to the experimental data and discussed in terms of the mechanism of drug release (analysis of the b and n values in the above equations). The carriers’ pH-dependent stability governed the drug release mechanism. Fast imatinib release from FeMIL-100 in the acidic endosomal buffer was due to matrix decomposition. The slower release from FeMIL-101-NH_2_ in both the endosomal and PBS media as well as from FeMIL-100 in PBS was due to the slow release from the micropores, because these materials are stable in pH-neutral solutions. The slowest release rate observed for MIL-100 was due to smaller pore entrances in MIL-100 compared with MIL-101-NH_2_.

Cellular cytotoxicity was determined based on several tests to provide comprehensive information on the mechanism of imatinib’s influence on neoplastic and healthy cells, human leukemia cells (HL60 line), and cardiomyocytes (undifferentiated H9c2 rat cells).

The viability and proliferation were determined using the PrestoBlue^TM^ test. For the IMB@FeMOF composites, the protective effect of the carrier increased with the drug concentration. At the highest concentration used (25 μmol/dm^3^), the viability of the cardiomyocytes decreased to 5% only when pure IMB was used, while it remained at an extremely high level (above 95%) when IMB was incorporated into an MOF. The imatinib released from the composites was quite effective against neoplastic cells. The imatinib released from the composites inhibited leukemia cell proliferation at concentrations greater than 2.5 µmol/dm^3^ and practically stopped proliferation at the highest applied concentration (25 μmol/dm^3^).

Cell damage was measured with a ToxiLight assay. For both composites, no toxic effect of IMB on the cardiomyocytes was observed, even when the drug concentration was increased tenfold, while the free imatinib induced cell death in a dose-dependent manner.

When comparing all the results for both the IMB@MIL-100 and IMB@MIL-101-NH_2_ systems, it is worth noting that the properties of both MOF materials were quite similar, except for the release kinetics. For IMB@MIL-100, imatinib was released rather quickly in the more acidic solution than in the pH-neutral environment. This is quite promising for targeted drug delivery because of the acidic conditions around cancerous cells. This feature shows quite clearly that FeMIL-100 should be preferably used as a carrier for imatinib.

Over the past 20 years, significant progress has been made in enhancing the potency and specificity of small-molecule inhibitors targeting protein and lipid kinases. Since the FDA approval of imatinib in 2001, more than 70 new drugs have been approved for the treatment of cancers and non-cancerous conditions [70]. Apart from imatinib, a few other drugs have also been commercially successful due to the ability of changing fatal and untreatable acute leukemia into a chronic disease with a high survival rate [71]. Targeted therapies with the use of small-molecule kinase inhibitors are believed to have a key role in cancer treatment. In both academic and industrial research, a promising avenue involves developing suitable carriers which release active substances within the tumor cell microenvironment [72]. These carriers protect the drug payload from premature degradation while safeguarding healthy cells against unintended drug effects.

Our experimental results demonstrate that iron-based metal-organic framework carriers, specifically MIL-100 and MIL-101-NH_2_, effectively mitigate the adverse effects of imatinib on cardiomyocytes while maintaining its efficacy against human leukemia cells. These MOFs hold promise as desirable therapeutic agents.

However, this represents just the initial step in a longer journey. Adhering to the fundamental principles of drug administration—right patient, right medication, right dose, right route, and right time—imposes specific constraints on final drug formulations. A simple two-component composite of the carrier and active substance serves as a starting point. Although oral administration remains ideal for long-term treatments (as seen with imatinib under the trade names Gleevec and Glivec), the next phase will involve developing basic infusion formulations. These formulations should minimize MOF matrix corrosion, possibly through additional coatings, to achieve stable nanodispersion. Addressing the observed (too fast) imatinib release at a neutral pH level is crucial. Rigorous in vitro (and eventually in vivo) stability and compatibility testing with plasma and blood are required.

On a different note, our investigations into selected FeMIL structures have already yielded a ready-to-use formulation for personalized tuberculosis pulmonary therapy (isoniazid@FeMIL-101-NH_2_) [40]. Additionally, these iron-based MOFs, due to their efficient MRI contrasting properties, hold promise for theranostics. The imatinib@FeMIL-101-NH_2_ composite, after suitable formulation, may represent a step toward personalized medicine enabled by theranostics.

## 4. Materials and Methods

### 4.1. Materials

Trimesic acid, 2-aminoterepthalic acid, dimethylformamide (DMF), iron(III) chloride hexahydrate, sodium hydroxide, iron(II) chloride tetrahydrate, dichloro-dihydrofluorescein diacetate (DCFH-DA), and imatinib (IMB) were purchased from Sigma Aldrich (Merck KGaA, Darmstadt, Germany) and used without further purification.

The H9c2 cell line, Dulbecco Modified Medium (DMEM), was acquired from ATTC (Manassas, VA, USA). TrypLE™ Express and fetal bovine serum (FBS) were obtained from Gibco (Thermo Fisher Scientific, Paisley, Scotland).

The HL60 cell line was purchased from Sigma Aldrich (Merck KGaA, Darmstadt, Germany).

The ToxiLight™ assay was acquired from Lonza (Basel, Switzerland), the PrestoBlue™ test was acquired from Invitrogen (Thermo Fisher Scientific, Paisley, Scotland), and the GSH/GSSG-Glo™ assay and Caspase3/7- Glo assay were acquired from Promega (Madison, WI, USA).

### 4.2. Synthesis of FeMOFs

The FeMIL-101-NH_2_ was prepared solvothermally in deoxygenated DMF (dimethylformamide, flushed with pure nitrogen gas), following the procedure published by Bauer et al. [45]. The linker, 2-aminoterephthalic acid (1 g), was dissolved in 120 g of DMF, forming a homogeneous solution. The second solution was prepared by dissolving 3.3 g of iron salt (Fe(NO_3_)_3_·6H_2_O) in 30 mL of DMF. The linker solution was then added dropwise to the iron nitrate solution and stirred continuously while flushing with nitrogen for 15 min. The mixture was then placed in a Teflon-lined steel autoclave and kept at 110 °C for 24 h. The brown product was filtered, washed with methanol (96%) for 3 days, and dried at room temperature overnight.

The FeMIL-100 was prepared according to its published procedure [73]. Trimesic acid (1.676 g) was dissolved in a 1 M solution of NaOH (23.72 g) and added dropwise to the aqueous solution (97.2 g) of iron(II) chloride tetrahydrate (2.26 g). The mixture was stirred at room temperature for 24 h. The obtained brown solid was filtered, washed with methanol (96%), and dried at 80 °C overnight.

### 4.3. Preparation of IMB@MOF Composites

Methanol-washed MOF samples were activated under a vacuum at 100 °C for 60 min to remove the solvent from the pores. Imatinib was loaded into the MOF matrix by mixing 2 mL of a saturated IMB solution in methanol with 100 mg of FeMIL-100 or FeMIL-101-NH_2_. The suspension was mixed for 12 h at room temperature, and the product was separated by centrifugation and washed twice with methanol. The obtained materials are denoted here as IMB@FeMIL-100 and IMB@FeMIL-101-NH_2_. The imatinib content was determined by UV-Vis spectrophotometry. Briefly, 10 mg of IMB@FeMIL-100 or IMB@FeMIL-101-NH_2_ was suspended in 50 mL of phosphate-buffered saline (PBS) and stirred for 7 days. After this time, supernatant samples (1 mL) were collected and centrifuged at 10,000 rpm and analyzed using a UV-Vis PerkinElmer Lambda spectrophotometer at a wavelength λ of 255 nm. PBS was used as a blank to determine the drug content from the calibration curve (linear graph). The percentage of drug content (DC%) in the MOF matrices was determined using the following formula (Equation (5)):

(5) $$\mathrm{DC}\%=\frac{\mathrm{weight}\;\mathrm{of}\;\mathrm{incroporated}\;\mathrm{drug}}{\mathrm{weight}\;\mathrm{composite}}\times 100\%$$

### 4.4. Characterization

The structure and crystallinity of the obtained MOFs and composites with the drug (FeMOFs) were examined through X-ray powder diffraction (XRD) using a Rigaku MiniFlex 600 (Rigaku, Wrocław, Polska; Testchem, Szarów, Polska) diffractometer in reflection mode using CuK_α_ radiation (ʎ = 0.154 nm). XRD patterns were collected with steps of 0.02°.

Nitrogen adsorption isotherms were determined by the standard method at the liquid nitrogen temperature using an ASAP 2025 (Micromeritics, Norcross, GA, USA) static volumetric apparatus. Before adsorption, the samples were outgassed at 100 °C using a turbomolecular pump overnight. The micropore volume was calculated from the isotherm desorption branch, and the BET area was determined using a t-plot.

The IR spectra were measured using Perkin Elmer Frontier (Shelton, CT, USA) equipped with a DTGS detector at a spectral resolution of 2 cm^−1^. For FT-IR measurements, the powder sample was deposited as a thin layer on a silicon wafer by evaporating a few drops of its suspension in methanol. The wafer was placed in an IR cell closed with KBr windows. Then, the sample was outgassed under a vacuum at 100 °C for 1 h to remove the residual methanol and water adsorbed from air. The spectra were normalized to the intensity of the maximum characteristic of the carbonyl C=O stretching vibration (1380–1390 cm^−1^).

Elemental analysis was performed using a CHNS (Vario Micro Cube, Elementar Analysensysteme GmbH, Langenselbold, Germany) elemental analyzer with an electronic microbalance.

SEM images were obtained using a Tescan Vega3 LMU (Tescan, Brno, Czech Republic) microscope with an LaB6 emitter (voltage of 30 kV). The samples were coated with gold prior to imaging.

### 4.5. In Vitro IMB Release Profiles

The drug release study was performed using the dialysis bag method (12–14 kDa MWCO). First, 20 mg of IMB@FeMIL-100 or IMB@FeMIL-101-NH_2_ was suspended in 5 mL of the release medium and transferred to a dialysis bag (SpectaPor tubing with a cutoff of 12,000 daltons (Sigma-Aldrich)). The dialysis bag was then placed in a conical tube containing 45 mL of the release medium and stirred at 200 rpm at 37 °C. The release was carried out at at neutral and slightly acidic pH levels, provided by phosphate buffer solution (PBS, pH = 7.4) and endosomal buffer (Endos, pH = 4.2). The concentration of imatinib released at 0.5, 1, 2, 3, 6, 8, 10, 24, and 48 h was analyzed using a UV-Vis PerkinElmer Lambda (Shelton, CT, USA) spectrophotometer at a wavelength λ of 255 nm. Origin 2018 software (OriginLab, Northampton, MA, USA) was used for fitting the release profiles.

### 4.6. Cell Cultures and Viability Assay

#### 4.6.1. Cell Cultures

Undifferentiated rat H9c2 cardiomyocyte-like cells were cultured in Dulbecco’s Modified Essential Medium (DMEM) supplemented with 10% (*v*/*v*) fetal bovine serum (FBS). HL60 human lymphoblast-like cells were maintained in RPMI-1640 medium supplemented with 20% (*v*/*v*) FBS. All cell lines were maintained until the confluence level reached 80% and then were passaged. Cell subculture or processing was performed by trypsinization with TrypLe™ Express or, in the case of HL60, by centrifugation. Between the third and seventh passages, the cells were seeded in a 96 well culture plate at a density of 10 × 10^3^ cells per well. After 24 h, the cells were exposed to the free drug and the drug released from MOFs at final drug concentrations of 1.25, 2.5, 5, 10, and 25 µmol/dm^3^. The effect of empty FeMOFs was evaluated by adding materials at five final concentrations: 1.2, 0.6, 0.3, 0.15, and 0.075 mg/mL. For all experimental procedures, the cells were maintained under optimal conditions at 37 °C, 5% CO_2_, and 95% humidity.

#### 4.6.2. Cell Viability and Cytotoxicity

The proliferation of cells and the cytotoxicity effect of the studied composites, free drug, and raw MOFs were determined by the ToxiLight™ assay. The assay kit was used to evaluate the concentration of adenylate kinase (AK) in the supernatant (corresponding to the number of damaged cells) and lysate (representing intact adherent cells) and was recalculated to the percent of toxicity according to the manufacturer’s protocol. Cell viability and metabolic capacity were examined using the resazurin-based reagent PrestoBlue™. The assay is based on the reduction of oxidized nonfluorescent blue resazurin to a red fluorescent dye (resorufin) by the mitochondrial respiratory chain in live cells.

#### 4.6.3. Mitochondrial Membrane Potential

The mitochondrial membrane potential was measured using a MitoTracker^®^ Red CMXRos assay (Thermo Fisher Scientific, Paisley, Scotland). The activity was measured after 24 h of cardiomyoblast incubation (H9c2 cell line). The test was performed according to the protocol provided by the manufacturer. In active mitochondria, the nonfluorescent probe is oxidized into its fluorescent form, and thus the measured intensity is proportional to the activity and mitochondria potential. The results were reported in relative fluorescent units ± standard deviation (SD) for 6 samples. Untreated cells were used as a control.

#### 4.6.4. Cell Morphology

The cell morphology for the control (not treated) and after incubation for 24 h in the presence of IMB, IMB@MIL-100, and IMB@FeMIL-101-NH_2_ was observed under a CKX53 inverted microscope (Olympus, Tokyo, Japan) after eosin and hematoxylin staining.

#### 4.6.5. Oxidative Stress and Superoxide Production

Imatinib-induced oxidative stress was measured using a GSH/GSSG-Glo^TM^ assay. Oxidative species produced by the cells were in contact for 24 h with the studied composites, free drug, and empty MOFs and were detected with a dichloro-dihydrofluorescein diacetate (DCFH-DA) assay. DCFH-DA was internalized within the cells in reduced form. In the presence of ROS, the probe oxidized into its fluorescent form, and thus the measured intensity was proportional to the intracellular oxidative stress. The results were reported as the percentage of the ROS level ± standard deviation (SD) for 6 samples. The cells treated with H_2_O_2_ (10 μmol/dm^3^) were used as a positive control group, and the untreated cells were a negative control.

## Figures and Tables

**Table 1:** Selected textural properties of the studied materials (Fe-MIL-100, FeMIL-101-NH_2_, and IMB@FeMIL-100 and IMB@FeMIL-101-NH_2_ composites) calculated based on low-temperature nitrogen adsorption and desorption.

Sample	S_BET_ (m^3^/g)	V_micro_ (cm^3^/g)
FeMIL-100	1413	0.54
IMB@FeMIL-100	50	0
FeMIL-101-NH_2_	969	0.38
IMB@FeMIL-101-NH_2_	23	0

**Table 2:** Basic parameters obtained by fitting Weibull (W) and Korsmeyer–Peppas (KP) release models: b and n values from Equations (2) and (4), Pearson’s r coefficients r, and residual sum of squares (RRS).

Parameter		IMB		IMB@FeMIL-100		IMB@FeMIL-101-NH_2_	
Solution		PBS	Endos	PBS	Endos	PBS	Endos
b value	W	0.59 ± 0.06	0.47 ± 0.19	0.82 ± 0.03	0.58 ± 0.15	0.75 ± 0.01	0.59 ± 0.02
n value k_m_ value	KP	0.42 ± 0.05 7.76 × 10^−3^	0.68 ± 0.30 2.00 × 10^−3^	0.71 ± 0.03 1.70 × 10^−5^	0.40 ± 0.09 7.59 × 10^−3^	0.60 ± 0.05 2.34 × 10^−4^	0.52 ± 0.03 8.13 × 10^−4^
Pearson’s r coefficient	W	0.995	0.916	0.996	0.966	0.999	0.995
	KP	0.992	0.928	0.997	0.973	0.993	0.998
RSS	W	0.002	0.055	0.083	0.023	0.019	0.039
	KP	0.001	0.022	0.016	0.008	0.009	0.002

## Data Availability

Data available from corresponding author upon request.
